# Anticorrosion and antimicrobial performance of extracted and fractionated phytochemicals of terminalia bentzoe leaves

**DOI:** 10.1038/s41598-025-08585-z

**Published:** 2025-07-09

**Authors:** N. A. El-Sawy, A. M. Fathi, S. K. Ali, D. A. Abdelrheem, M. M. Hegab, M. M. El-Deeb

**Affiliations:** 1 Chemistry Department of Medicinal and Aromatic Plants, Research Institute of Medicinal and Aromatic Plants, Beni-Suef University, Beni-Suef, Beni-Suef, 62514 Egypt; 2 Physical Chemistry Department, National Research Centre, 33 El Bohoth St. (El-Tahrir St. Former), P.O.12622, Dokki, Giza Egypt; 3 Department of Agricultural Microbiology, Faculty of Agriculture, Beni-Suef University, Beni-Suef, Beni-Suef, 62521 Egypt; 4 Chemistry Department, Faculty of Science, Beni-Suef University, Beni-Suef, Beni-Suef, 62511 Egypt; 5 Botany and Microbiology Department, Faculty of Science, Beni-Suef University, Beni-Suef, Beni-Suef, 62511 Egypt

**Keywords:** Terminalia bentzoe leaves, Extraction, copper protection, Antimicrobial activity, Chemistry, Materials science

## Abstract

Methanolic extraction (ME) of terminalia bentzoe leaves and its aqueous (QF) and n-butanol (nBF) fractions are investigated as sustainable green compounds for both anticorrosion and antimicrobial activity. Extracted and fractionated phytochemicals and their functional groups are detected by GC-MS and FTIR. According to the electrochemical results, ME, QF and nBF samples are considered as anodic inhibitors that mainly inhibit the anodic dissolution of copper (Cu) in 0.6 M NaCl solution. Their inhibition efficiencies increase by increasing their concentrations up to 60 ppm, and the highest value of inhibition efficiency is found to be 94.4% for nBF. The presence of the protective adsorbed layer from the extracted and fractionated phytochemical compounds onto the Cu surface was supported by FESEM analyses and theoretical. On the other hand, exploring the antimicrobial activity of ME, QF and nBF samples against Staphylococcus aureus, Bacillus cereus, Listeria monocytogenes, Escherichia coli, Salmonella typhi, Shigella boydii, Aspergillus niger and Candida albicans was made by using modified Kirby-Bauer disc diffusion technique. Data indicated good resistance against a variety of bacterial infections for the studied green compounds.

## Introduction

The extensive use of copper and its alloys in a variety of industrial applications, including decorations, electronics, building, industrial equipment, and transportation gave them an opportunity in many studies^1,2^. However, because of the use of high chloride ions in some aggressive conditions, particularly in a marine environment, copper can severely corrode^3^. Thus, copper corrosion protection has drawn the attention and several efforts have been devoted to develop an eco-friendly and effective copper corrosion inhibitors, including imidazoles, triazoles, and some schiff bases with N, O, S, P, polar functional groups, and/or conjugated double bonds^4–7^. On the other hand, plant extracts have received much attention, because they have anti-microbial and anti-corrosion properties. These properties can be explained, due to richness in bioactive compounds. The phytochemicals included in plant extracts have electron-rich regions and functional groups that enable them to adsorb powerfully on metal surfaces with very low concentrations. These phytochemicals contain tannins, phenolics, organic acids, amino acids, alkaloids, and flavonoids, which can be employed to prevent metallic components from corroding^8,9^. Many efforts have been occured recently to report on plant extracts’ ability to suppress metal corrosion growth^10–14^. Many studies show the inhibition effects of various plant extracts against the corrosion of Cu in acidic^15–20^ and saline solutions^21–27^.

Terminalia bentzoe is one of the medical plants that classified as the second largest genus in the Combretaceae, which is widespread in Egypt and other sub-tropical and tropical regions of the world. It has been reported that the plant extract of Terminalia is commonly rich in phenolics, flavonoids, alkaloids, triterpenoids, tannins and other compounds^28^. Because of these phytoconstituents, most of Terminalia species have multiple biological, pharmacological and medicinal activities^29–35^.

The main aim of this study is to examine the usage of the methanolic Terminalia bentzoe extract and its aqueous and n-butanol fractions as anti-corrosion and anti-microbial properties. Thus, the present work studies the extraction (methanolic), fractionation (aqueous and n-butanol) and elucidation of the Terminalia bentzoe leaves, and investigates their extracted and fractionated phytochemicals samples as sustainable green inhibitors against Cu corrosion in saline solution, using electrochemical measurements, surface analysis, and Monte Carlo (MC) simulation. In addition, modified Kirby-Bauer disc diffusion technique is used to inspect their effect as antimicrobial compounds against Staphylococcus aureus, Bacillus cereus, Listeria monocytogenes, Escherichia coli, Salmonella typhi, Shigella boydii, Aspergillus niger and Candida albicans.

## Experimental

### Materials and chemicals

Collected Terminalia bentzoe leaves from Giza Zoo, Cairo, Egypt and identified at the Ministry of Agriculture, Egypt was used in the study. Sodium chloride, methanol, n-Butanol were purchased from PIOCHEM laboratory chemicals Co., Egypt. The working electrode was made from Cu metal and the stock solutions of the studied compounds were prepared using distilled water.

### Extraction, fractionation and chemical constituents

**Fig. 1:**
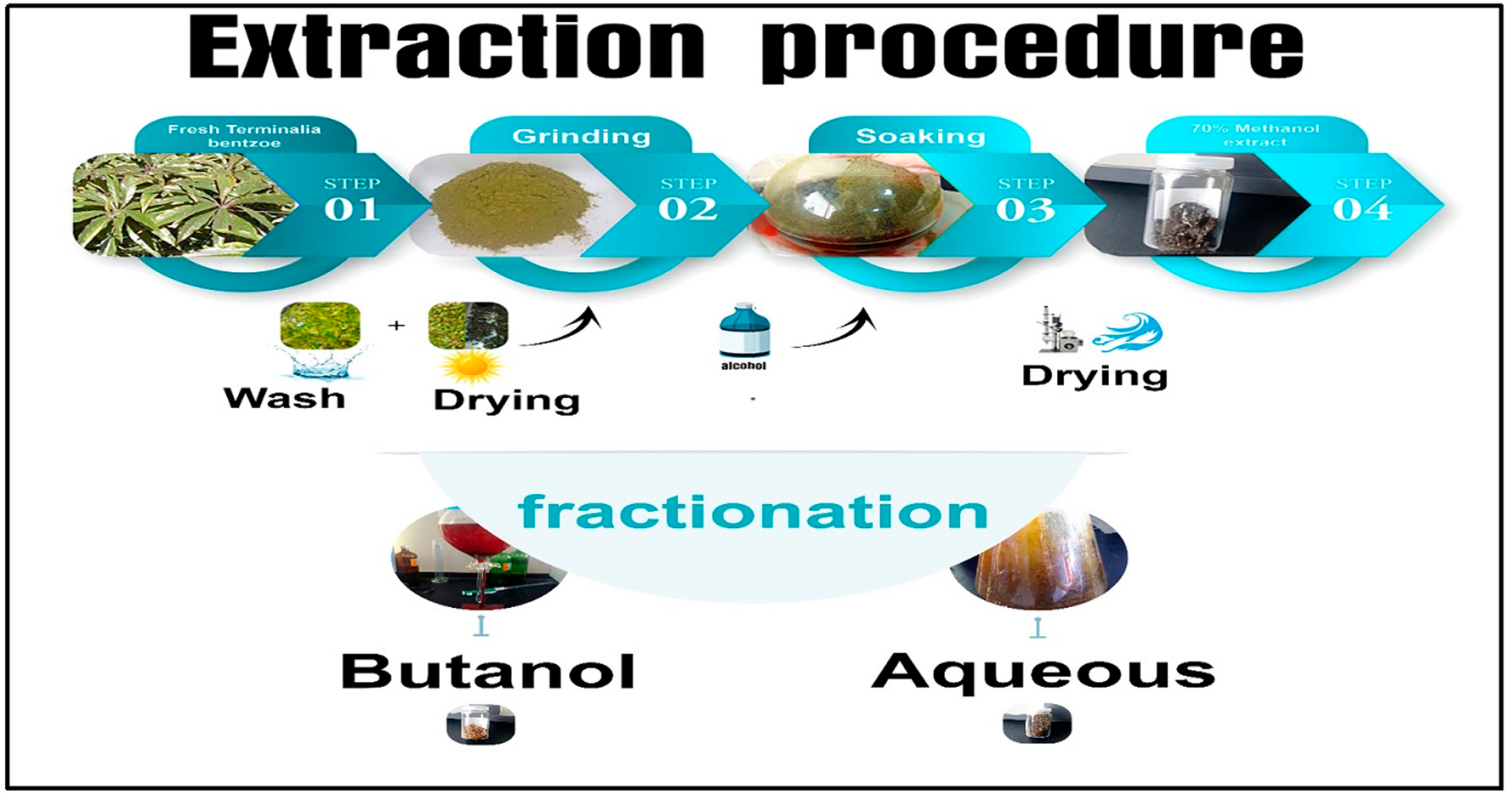
Schematic diagram represents the extraction and fractionation processes of terminalia bentzoe leaves.

Fresh leaves were washed 3 times with 10 L of distilled water, then dried at room temperature for 3 days, and grounded for 4 h prior to extraction. 710 g of dried and grounded sample were thoroughly extracted with 10 L of 70% methanol at room temperature with constant shaking. 43 g of the whole extract was obtained after the vaporization of the solvent under reduced pressure. Initially, 38 g of the total extract is dissolved in 150 mL distilled water and then extracted with 150 mL of n-Butanol. Finally, the mixed extractions were dried under vacuum to produce 5.7 g sample from n-Butanol fraction and 12.2 g sample from aqueous fraction. Extraction and fractionation were graphically represented in Fig. 1.

The chemical composition of methanol extract (ME), aqueous fraction (QF) and n-butanol fraction (nBF) samples were performed using Trace GC-TSQ mass spectrometer (Thermo Scientific, Austin, TX, USA) with a direct capillary column TG–5MS (30 m × 0.25 mm × 0.25 μm film thickness). Initially, the temperature of column oven was adjusted at 50 °C, and then raised by 5 °C /min to 250 °C to be held for 2 min. Finally, the temperature raised to 300 °C by 30 °C /min and held for 2 min. The injector and MS transfer line temperatures were kept at 270 and 260 °C respectively. Helium was used as a carrier gas at a constant flow rate of 1.0 mL/min. The solvent delay was 4 min, and 1 µl of diluted samples are automatically injected by Auto sampler AS1300 coupled with GC in the split mode. Over the range of m/z 50–650 in full scan mode, EI mass spectra were collected at ionization voltages of 70 eV. The ion source temperature was fixed at 200 °C. Identification of the components was done by comparing their mass spectra with those of WILEY 09 and NIST 14 mass spectral database. Infrared measurements were passed out using a Bruker optics VERTEX 70 spectrophotometer, Germany.

### Electrochemical experiments

Electrochemical tests in the absence and presence of ME, QF and nBF samples in 0.6 M NaCl were performed in a typical three-electrode configuration cell consisting of Cu (1.0 cm^2^) as working electrode (WE), saturated Ag/AgCl as reference electrode (RE), and counter electrode (CE) from Pt wire using Origalys OGS 200 potentiostat/galvanostat, France.

Potentiodynamic polarization curves were adjusted at a potential range between − 0.3 and 0.1 V (vs. Ag/AgCl) with scan rate of 1 mVs^− 1^. Electrochemical impedance spectroscopy (EIS) experiments were implemented at E_OCP_ with AC voltage amplitude of 10 mV and a scanning frequency of 100 kHz to 0.1 Hz. The electrochemical measurements were recorded and fitted by OrigaMaster-5 software. Before each experiment, Cu working electrode was polished with fine grade emery paper (1200P then 2500P), cleaned, rinsed with distilled H_2_O, and lastly dried before each experiment.

### Surface analysis

Morphological structure for the Cu samples after 3 days of immersion in 0.6 M NaCl in the absence and presence of 60 ppm of each of ME, QF and nBF samples were analyzed using scanning electron microscope JEOL-JSM-5600, Japan.

### Monte Carlo simulation

Monte Carlo (MC) simulation was employed to determine the adsorption energy (ΔEads) of ME, QF and nBF samples on Cu box with a size of 10 nm (containing 994 atoms of copper) using Materials Studio 7 package^36^. The copper unit cell adopted a face-centered cubic structure with a length of 4.022 Å, possessing space group symmetry Fm-3 m, and crystal class m-3 m. The structural optimization is performed using the Condensed-Phase Optimized Molecular Potential for Atomistic Simulation Studies (COMPASS II) force field^37,38^. The convergence criteria for energy, force, stress, and displacement are set to 2.0 × 10^− 5^ kcal·mol^− 1^, 0.001 kcal·mol^− 1^·Å^−1^, 0.001 GPa, and 1.0 × 10^− 5^ Å, respectively. Both Ewald and atom-based methods were employed to handle electrostatic and van der Waals forces. The optimization process was executed using the Forcite Module. The MC simulation utilized the Adsorption Locator Module, based on simulated annealing with 10 temperature cycles and 100,000 Monte Carlo steps per cycle. The maximum and final temperatures were set to 1.0 × 10^5^ K and 100 K, respectively. The Adsorption Locator Module utilizes the Metropolis MC method to seek the lowest-energy adsorption configurations. The construction of ME, QF and nBF samples to investigate their adsorption on Cu surface was designed according to the percentage of their phytochemical compounds derived from GC-MS analysis as presented in Table 1.

**Table 1:** GC-MS analysis for the main phytochemicals’ compounds presented in the studied ME, QF and nBF sample.

No	Name	Chemical formula	RT	Area%	Sample
1	9-Octadecenamide (Z)	C_18_H_35_NO	34.24	50.24	ME
			34.56	24.15	nBF
			34.27	17.34	QF
2	E, E,Z-1,3,12-Nonadecatriene-5,14-diol	C_19_H_34_O_2_	40.48	12.96	ME
			40.40	12.59	nBF
			–	–	QF
3	10-Octadecenoic acid, methyl ester	C_19_H_36_O_2_	29.58	11.41	ME
			–	–	nBF
			29.59	6.03	QF
4	1,2-Benzenedicarboxylic acid	C_24_H_38_O_4_	36.76	8.14	ME
			–	–	nBF
			36.76	7.92	QF
5	Hexadecanoic acid, 1-(hydroxymethyl)-1,2-ethanediyl ester	C_35_H_68_O_5_	27.31	3.47	ME
			27.24	3.14	nBF
			–	–	QF
6	Linoleic acid ethyl ester	C_20_H_36_O_2_	30.60	3.30	ME
			29.44	0.37	nBF
			30.66	1.58	QF
7	2-Hydroxy-3-[(9E)-9-Octadecenoyloxy]Propyl (9E)-9-octadecenoate	C_39_H_72_O_5_	30.54	1.29	ME
			32.94	4.31	nBF
			–	–	QF
8	Deoxyspergualin	C_17_H_37_N_7_O	31.10	5.62	ME
			31.07	5.61	nBF
			–	–	QF
9	9-Octadecenoic acid (Z)-, 2-hydroxy-1-(hydroxyl methyl)ethyl ester	C_21_H_40_O_4_	30.78	1.39	ME
			–	–	nBF
			30.79	3.45	QF
10	3’,8,8’-Trimethoxy-3-piperidyl-2,2’-bi-naphthalene-1,1’,4,4’-tetrone	C_28_H_25_NO_7_	36.76	8.14	ME
			36.76	7.92	nBF
			36.75	4.63	QF

### Antimicrobial activity

#### Bacteria testing

Gram-positive bacteria (*Staphylococcus aureus*, *Bacillus cereus*, and *Listeria monocytogenes*) and Gram-negative bacteria (*Escherichia coli*, *Salmonella typhi*, and *Shigella boydii*) were cultivated in nutritious broth for 24 h before testing for antibacterial activity. *Aspergillus niger* and *Candida albicans* were cultivated in potato dextrose broth for 48 h before using for antifungal activity. Using a modified Kirby-Bauer disc diffusion technique, the widths of the inhibition zones were determined in millimeters to determine the antibacterial activity of the examined samples^39,40^. Filter discs impregnated with water served as a negative control for antimicrobial activity, while Gentamycin (antibacterial agent) and nystatin (antifungal) standard discs served as positive control.

#### Determination of the minimum Inhibition concentrations (MICs)

Terminalia bentzoe extract and its fractions revealed significant antimicrobial activity in antimicrobial susceptibility tests. Using dimethyl sulfoxide (DMSO) as a solvent, the condensed extract was utilized to generate a stock solution of 10 mg/mL. The solution was diluted to achieve concentrations ranging from 3.12 to 50 mg/mL.

## Results and discussions

### Structural elucidation of of ME, QF and nBF samples

GC-MS analysis of ME, QF and nBF samples are performed using Trace GC-TSQ mass spectrometer, the retention time (RT), concentrations (% peak area) and molecular formula for the main phytochemicals presented in the studied samples are presented in Table 1.

**Fig. 2:**
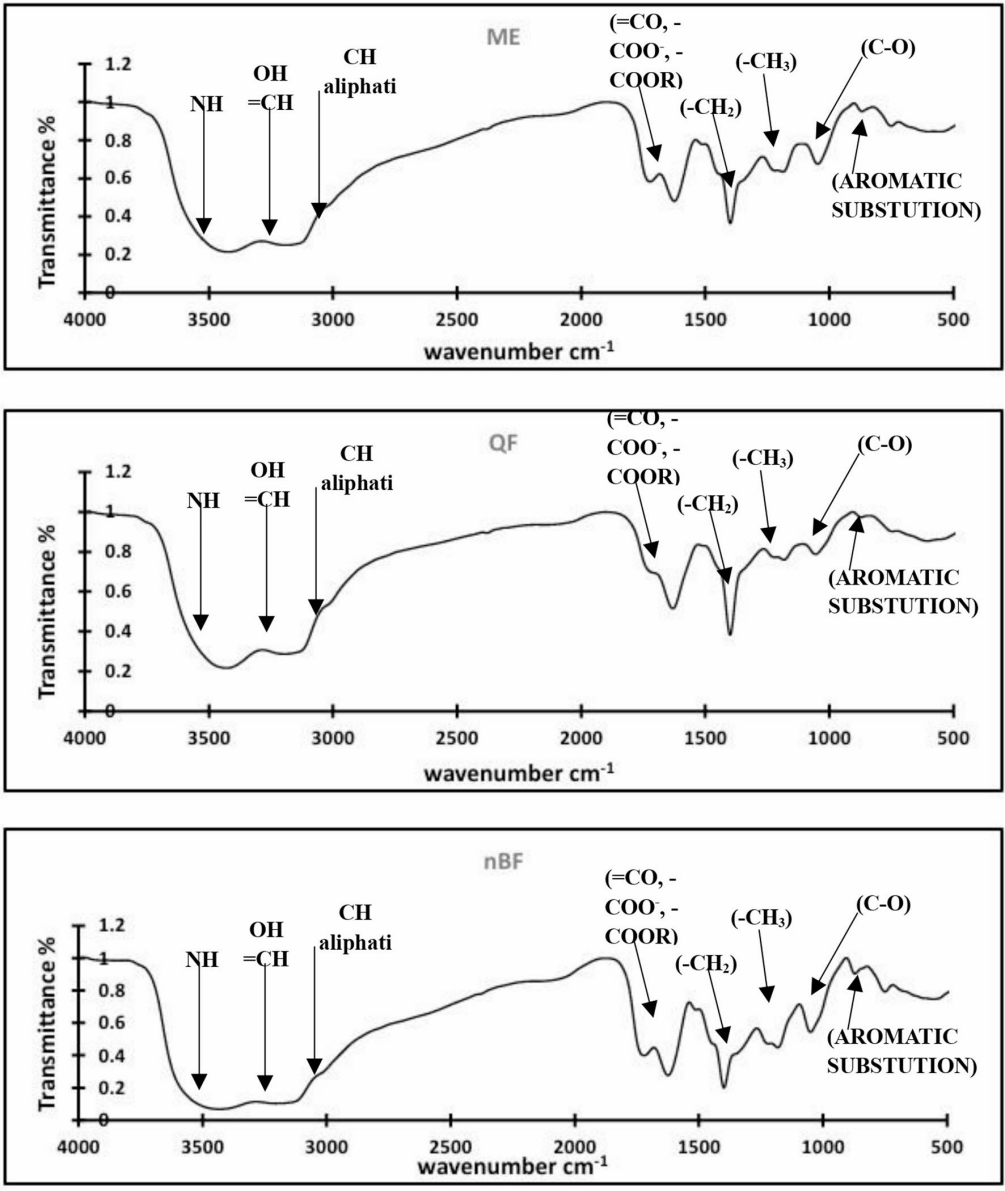
FTIR analysis of ME, QF and nBF.

Functional groups of the phytochemical active compounds presented in the studied ME, QF, and nBF samples are indicated using FTIR analysis as shown in Fig. 2. From the figure, it can be concluded that there is no drastic changes in the band positions, but some changes in the band intensities can be observed. The variation of the band intensities indicates the consistent percentages in the studied samples. Hence, the increase of transmittance means the decrease of the consistent construction. The infrared characteristic absorption bands and the transmittance for stretching and bending vibrations for C-H band modes, -COO- band modes, C-C band modes, CO-NH band modes and their assignment are summarized in Tables 2, 3, 4, 5 and 6).

**Table 2:** Assignments of the different stretching C-H band modes.

Wave number (Cm^− 1^)	Transmittance			Assignments
	ME	*n*-BF	QF	
3080^b^	0.149	0.191	0.422	Symmetric stretching vibration for CH present in vinyl (-CH = CH-) group
2964^b^	0.330	0.375	0.626	The symmetric stretching vibrations of C-H present in CH_3_ group
2920^b^	0.396	0.441	0.683	The symmetric stretching vibrations of C-H present in CH_2_ group
2851^b^	0.435	0.480	0.716	Asymmetric stretching vibrations of CH_2_ group
2839^sh^	0.480	0.525	0.749	The asymmetric stretching vibrations of C-H present in CH_3_ group

**Table 3:** Assignments of the different bending C-H band modes.

Wave number (Cm^− 1^)	Transmittance			Assignments
	ME	*n*-BF	QF	
1458^sh^	0.454	0.499	0.736	Bending deformation (scissoring mode) for CH_2_ group
1443^b^	0.421	0.466	0.708	
1390^b^	0.182	0.227	0.432	Bending deformation (scissoring mode) for CH_3_ group
1369^sh^	0.338	0.383	0.656	
930 ^sh^	0.907	0.952	0.983	The rocking vibration of CH_3_ group
833^m^	0.892	0.937	0.979	Out-of-plane deformation of CH group in 1,2-disubstituted benzene ring
750^m^	0.743	0.788	0.901	
698^sh^	0.764	0.809	0.892	δ (CH_2_) coupled with skeletal stretching vibrations

**Table 4:** Assignments of the different bending COO- band modes.

Wave number (Cm^− 1^)	Transmittance			Assignments
	ME	*n*-BF	QF	
3330^b^	0.051	0.096	0.284	Symmetric stretching of hydrogen bonded OH group in carboxylic acid
1730^m^	0.355	0.400	0.707	Symmetric stretching vibrations of (C=O) group in carboxylic and ester groups
1177^m^	0.402	0.447	0.761	Symmetric stretching for (C-O-C) group
1157^sh^	0.482	0.527	0.796	
840^w^	0.887	0.932	0.980	Deformation vibrations (C-O-C) and C-C-O groups
685^m^	0.751	0.796	0.885	

**Table 5:** Assignments of the different bending C-C band modes.

Wave number (Cm^− 1^)	Transmittance			Assignments
	ME	*n*-BF	QF	
1065^m^	0.517	0.562	0.789	Stretching vibration of (C-C) of planer zig-zag chain backbone
1620^s^	0.229	0.274	0.528	Stretching vibration of (C = C) in benzene ring

**Table 6:** Assignments of the different bending CO-NH band modes.

Wave number (Cm^− 1^)	Transmittance			Assignments
	ME	*n*-BF	QF	
3447^b^	0.019	0.064	0.218	Asymmetric stretching for NH in NH_2_ group
3227^b^	0.055	0.099	0.288	
1524^w^	0.648	0.693	0.834	The symmetric stretching vibrations of (C = O) group in amide
1215^sh^	0.423	0.468	0.772	The symmetric stretching vibrations of (C-N) group in amide

### Corrosion studies

#### Potentiodynamic polarization (PDP) measurements

PDP measurements are performed for Cu in 0.6 M NaCl solution in the absence and presence of various concentrations from 20 to 100 ppm of nBF, ME, and QF samples from – 0.3 to 0.1 V (vs. Ag/AgCl) with scan rate of 1 mVs^− 1^ at ambient temperature. The electrochemical polarization parameters are calculated and tabulated in Table 7. The inhibition efficiency (*IE*%) is estimated using the following equations^41^.

1 $$\:IE\%=\:\theta\:\:\times \:100=\left[\:{i}_{corr}^{o}-{i}_{corr}/{i}_{corr}^{o}\right]\times \:100$$

Where θ represents the surface coverage, $\:{i}_{corr}^{o}\:$and $\:{i}_{corr}$ denote the corrosion current densities for the blank solution and the studied compounds, respectively.

**Table 7:** Electrochemical parameters of Cu oxidation in 0.6 M NaCl in the absence and presence of different concentrations of ME, QF and nBF at ambient temperature.

Sample	Concn. of inhibitor, ppm	b_a_ mV dec^− 1^	b_c_ mV dec^− 1^	E_corr_, mV	I_corr_ , mA cm^− 2^	Corros. rate µm year^− 1^	*R*, KΩ cm^2^	IE %
Blank	0	82.7	– 45.4	– 220	0.0180	189.5	0.56	
nBF	20	23.5	– 28.4	– 175	0.0030	50.2	1.72	83.3
	40	25.4	– 32.1	– 175	0.0027	46.5	1.82	85.0
	60	22.9	– 35.1	– 144	0.0010	13.5	4.86	94.4
	80	28.7	– 40.3	– 192	0.0045	62.9	1.36	75.0
	100	17.5	– 20.5	– 205	0.0043	61.3	1.02	76.1
ME	20	18.7	– 24.7	– 184	0.0044	49.6	1.07	75.6
	40	35.9	– 52.6	– 186	0.0039	45.0	1.45	78.3
	60	34.5	– 37.9	– 172	0.0030	40.9	1.48	83.3
	80	23.4	– 27.4	– 177	0.0053	64.2	0.97	70.6
	100	30.8	– 35.6	– 175	0.0083	102.1	0.01	53.9
QF	20	18.2	– 17.3	– 197	0.0047	32.3	1.68	73.9
	40	30.7	– 44.6	– 154	0.0051	61.6	0.95	71.6
	60	25.0	– 31.5	– 185	0.0060	72.9	0.76	66.1
	80	44.0	– 39.6	– 198	0.0065	71.7	0.69	63.9
	100	21.8	– 26.4	– 213	0.0089	100	0.64	50.6

**Fig. 3:**
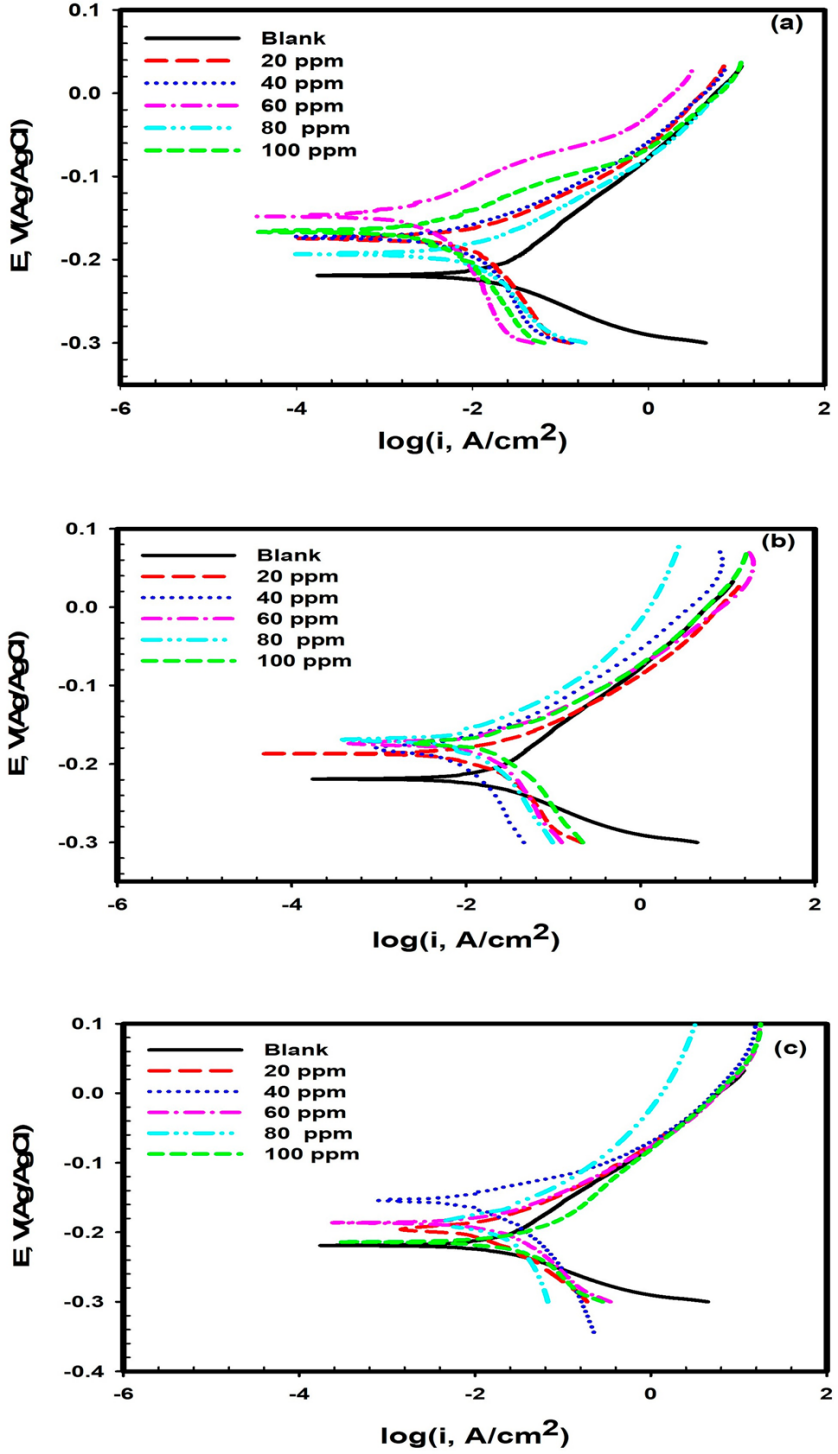
Potentiodynamic polarization of copper in 0.6 M NaCl in the absence and presence of different concentrations of (**a**) nBF, (**b**) ME, and (**c**) QF at ambient temperature.

Figure 3a–c represents the PDP of Cu in 0.6 M NaCl blank (free from inhibitors) and with different concentrations of ME, QF and nBF samples at ambient temperature. Results of Table 7 indicate that, the addition of different concentrations (20–100 ppm) of ME, QF, and nBF samples to 0.6 M NaCl solution shifts the corrosion potential (*E*_*corr*_) of Cu to more noble values, which agrees with the open circuit measurements, and reduces its corrosion current densities (*I*_*corr*_) compared to the blank solution, indicating that the ME, QF and nBF samples are considered as anodic inhibitors that mainly inhibit the anodic dissolution of Cu. This effect is interpreted bases of the adsorption of the phytochemical compounds in ME, QF and nBF samples on the anodic site of the Cu surface which forms a barrier adsorbed layer that blocks the active anodic sites in Cu surface. On the other hand, the values of the calculated IE% of the studied compounds increase with increasing their concentrations with the following order: QF ˂ ME ˂ nBF samples, and the maximum value is found to be 94.4% for 60 ppm of nBF. This order can be correlated to the orientation of the adsorbed phytochemical active compound on Cu surface, which will be discussed in MC simulation section. The mechanism of the electrochemical anodic dissolution of Cu and cathodic reduction of O_2_ in saline solution is summarized as follows^42,43^:

*Anodic dissolution of copper*.

(I) 2 $${\text{Cu }} \leftrightarrow {\text{ Cu}}^{ + } + {\text{ e }}^-$$
  3 $${\text{Cu}}^{ + } + {\text{ 2Cl}}^{ - } \leftrightarrow {\text{CuCl}}_{{\text{2}}} ^{ - }$$
(II) 4 $${\text{Cu }} + {\text{ Cl}}^{ - } \leftrightarrow {\text{ CuCl }} + {\text{ e}}^{ - }$$
  5 $${\text{CuCl}} + {\text{ Cl}}^{ - } \leftrightarrow {\text{CuCl}}_{{\text{2}}} ^{ - }$$
  6 $${\text{2 CuCl}}_{{\text{2}}} - {\text{ }} + {\text{ OH}}^{ - } \leftrightarrow {\text{Cu}}_{{\text{2}}} {\text{O }} + {\text{ 4Cl}}^{ - } + {\text{ H}}^{ + }$$

*Cathodic reduction of oxygen*.

7 $${\text{O}}_{{\text{2}}} + {\text{ 2H}}_{{\text{2}}} {\text{O }} + {\text{ 4e}}^{ - } \leftrightarrow {\text{ 4OH}}^{ - }$$

Reactions (2–3 & 4–5) are two possible cases of the initial electro-dissolution of copper. However, the presence of ME, QF and nBF molecules replace the adsorbed chloride ions which are originally adsorbed at the metal/solution interface and form a protective adsorbed layer that slows down copper dissolution.

### Electrochemical impedance spectroscopy measurements

**Fig. 4:**
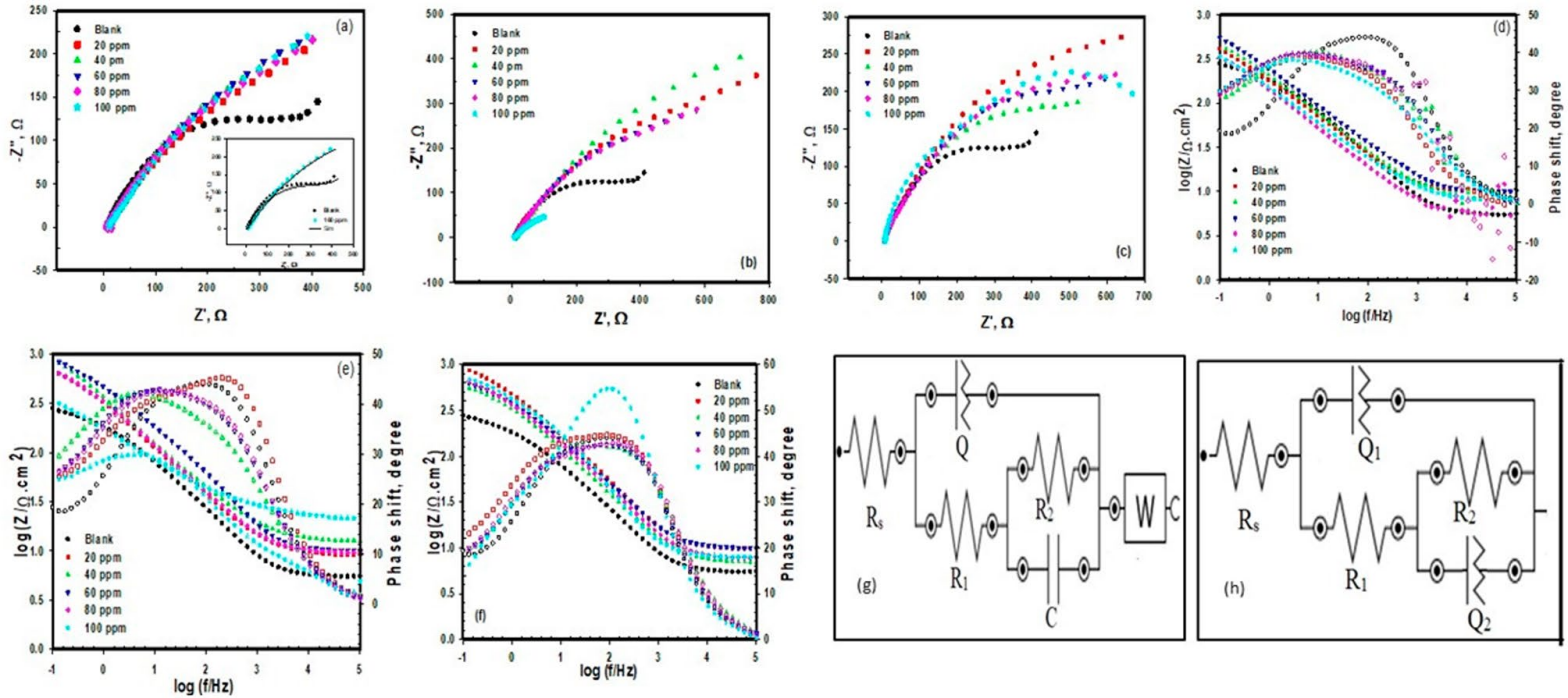
Nyquist and Bode plots for copper in 0.6 M NaCl with different concentrations of (**a**, **c**) nNB, (**b**, **e**) ME, and (**c**, **f**) QF, respectively. (**g**, **h**) equivalent circuit for blank and blank with inhibitors.

Nyquist plots of Cu in 0.6 M NaCl solution at E_ocp_ in the absence and presence of different concentrations of ME, QF and nBF samples are represented in Fig. 4. Nyquist spectra obtained consist of one depressed capacitive loop (Fig. 4a–c); the depression of the semicircle signifies on the inhomogeneity of the surface^44^. The diameter of the capacitive loop increased in the presence of the studied green compounds compared to the blank solution indicating their inhibitive effect of Cu corrosion. The high frequency capacitive loop can be recognized to the redox of Cu to Cu^+^ reaction, reflecting on the rate-determining phase in the charge transfer reaction during the corrosion process. The semicircle shapes for the uninhibited and inhibited copper are similar, which suggests the same electrochemical behavior. In addition, increasing the concentrations of these green compounds leads to increasing the diameters of the capacitive semicircles, because of changing the Cu/NaCl interface structure by substituting water molecules, producing barrier protective insulating layers adsorbed on the Cu surface. On the otherwise lowering the local dielectric constant and/or increasing the thickness of the adsorption barrier protection layer impede the charge transfer process across the interface.

The Bode plots in Fig. 4d–f show one phase with narrow maximum for copper in 0.6 M NaCl without inhibitors (Blank) indicating less protective layer on the copper surface despite the maximum broaden by adding the inhibitor and increasing its concentration indicating the formation of more protective film^45,46^. The results of the blank were fitted by equivalent circuit shown in Fig. 4g that consists of two parallel simple circuits in series with the solution resistance (R_s_); the first one involves a resistance for the polarization (R_1_) which included the charge transfer resistance through the interface of copper surface and the solution (R_ct_), the resistance of the diffused layer (R_d_), and the resistance of any corrosion products accumulated at the surface^47,48^. R_1_ was in parallel with the constant phase element (CPE), Q. The second circuit consists of the resistance (R_2_) and the double layer capacitance (C) of the oxide film formed on the copper surface. A diffusion process occurs on the copper surface that represented by a Warburg impedance, Z_w_, combined to R_2_C combination for the for copper ions diffusion through corrosion^49^. The presence of Warburg impedance indicated that the corrosion process is controlled by both the charge transfer and the diffusion.

The experimental results of the inhibited copper was fitted by an equivalent circuit (Fig. 4h) which is like the one used for the blank but the combination R_2_C is replaced by R_2_Q_2_ for the adsorbed layer of the extract molecules without the presence of the diffusion. The impedance of the phase constant Q can be described by both the modulus of CPE, Y_o_, and the parameter of the phase shift deviation, n, as follows;^50,51^.

$${\text{Z}}_{{{\text{CPE}}~~}} = {\text{ 1}}/{\text{Y}}_{{\text{o}}} \left( {{\text{j }}\omega } \right)^{{\text{n}}}$$

ω, and j is the angular frequency, and the imaginary number, respectively. The fitted parameters for both inhibited and uninhibited copper are given in Table 8. R_2_ increases by increasing the inhibitor concentration to a certain concentration which is 60 ppm for nBF and ME and 20 ppm for QF. With the increase in the resistance, a decrease in Y occurs, which indicates an increase in the adsorbed layer thickness. In addition, the presence of inhibitors results in increasing the value of n which means increasing the surface homogeneity as a result of the inhibitor molecules’ adsorption on the active sites^52^.

**Table 8:** The impedance parameters for copper in 0.6 M NaCl in the absence and presence of different concentrations of ME, QF and nBF at ambient temperature.

Type of inhibitor	Conc. (ppm)	*R*_s_ (Ω)	*R*_1_ (Ω cm^2^)	Q		*R*_2_ (KΩ cm^2^)	C (µF cm^− 2^)	W mΩ^−1^
				Y (µΩ^−1^cm^−2^)	*n*			
Blank	0	8.37	0.067	762	0.54	0.399	11	10.4

				Q_1_			Q_2_	
				Y(mΩ^**−1**^**cm**^**−2**^**)**	*n*		Y (mΩ^**−1**^**cm**^**−2**^**)**	*n*
nBF	20	7.41	8.95	1.83	0.50	1.05	0.029	0.83
	40	7.72	5.12	1.78	0.50	1.11	0.018	0.85
	60	7.00	4.31	1.72	0.52	1.12	0.012	0.90
	80	6.12	8.95	1.69	0.50	1.03	0.019	0.80
	100	9.84	9.72	1.79	0.50	1.01	0.021	0.78
ME	20	9.72	559	0.38	0.64	0.89	1360	0.70
	40	13.3	296	0.39	0.70	0.99	1010	0.65
	60	8.73	226	0.62	0.63	1.04	619	0.69
	80	9.21	223	0.54	0.64	0.76	799	0.59
	100	8.11	118	4.40	0.55	0.68	2510	0.59
QF	20	7.81	26.70	0.093	0.83	0.99	0.92	0.50
	40	6.91	26.80	0.14	0.78	0.79	0.91	0.50
	60	9.10	29.10	0.19	0.70	0.76	0.58	0.50
	80	7.80	147.10	0.27	0.70	0.75	0.75	0.60
	100	8.00	227.00	0.16	0.80	0.73	1.01	0.58

The results for impedance measurements agree with those observed from the polarization measurements which indicate the ability of the three extracts to decrease the corrosion of copper in NaCl solution.

#### Time of immersion effect on the corrosion behaviour

**Fig. 5:**
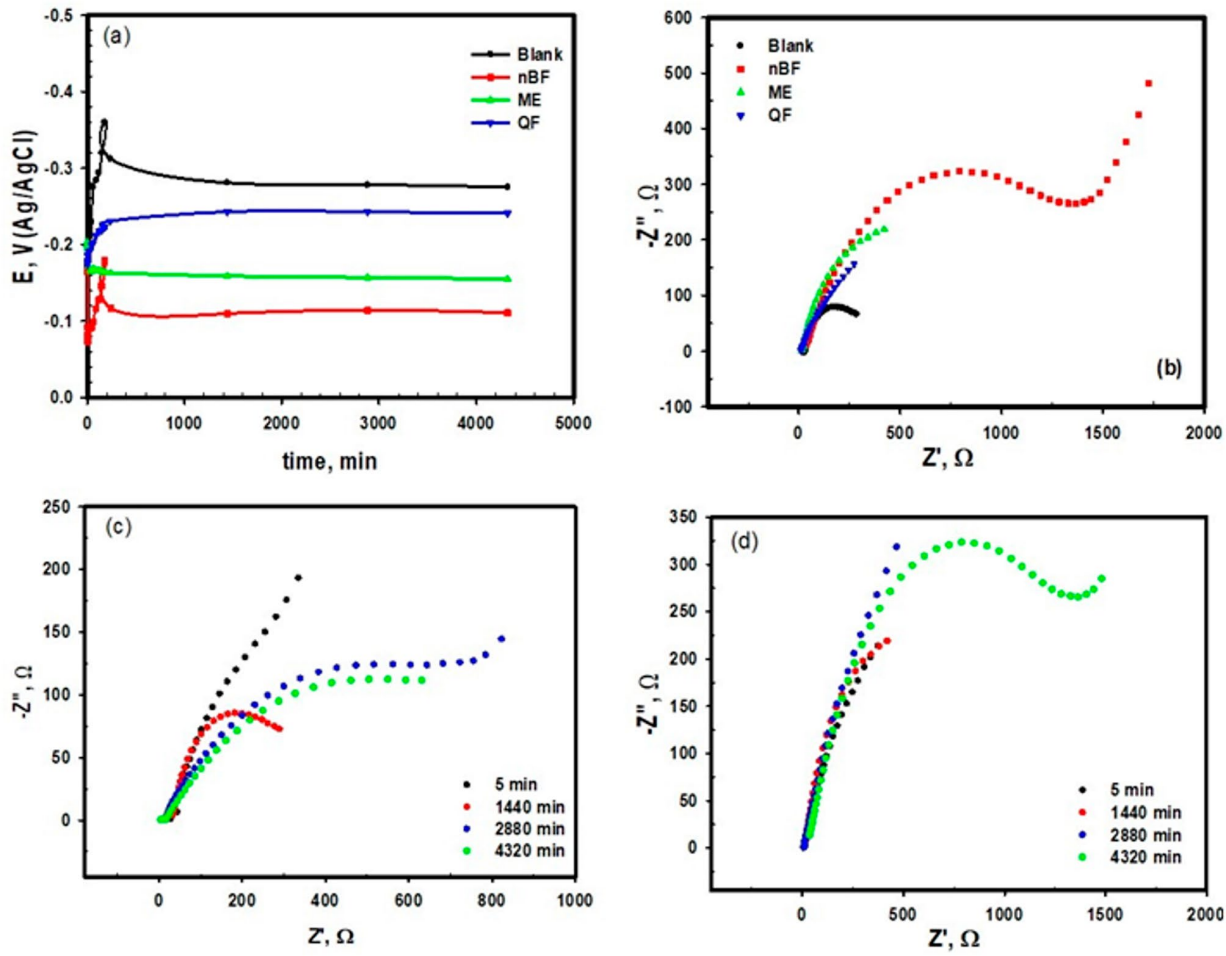
Open circuit measurements (**a**), Nyquist plots (**b**) for copper after 3 days of immersion in 0.6 M NaCl in the absence and presence of 60 ppm of nBF, ME, and QF, (**c**, **d**) Nyquist plots for copper at different time of immersion in 0.6 M NaCl solution, and 0.6 M NaCl solution containing 60 ppm of nBF, respectively.

Time of immersion effect on the corrosion behavior of copper was tracked through the relation between the open-circuit potentials (E_ocp_ values) and the time for 3 days in 0.6 M NaCl solution free from (blank) and contains 60 ppm of ME, QF and nBF samples and showed in Fig. 5a. After 240 min of immersion in the inhibited solution, the steady state potential nearly was grasped; however, in the uninhibited solution, it took more than 1000 min to reach. The potential of steady state is shifted to either more negative or more positive in the presence of the inhibitors depending on the type of the extract and its composition.

The value of open-circuit potential for the uninhibited copper after long time (3 days) of immersion in 0.6 M NaCl is – 280 mV, however the E_ocp_ values for inhibited samples by ME, QF and nBF samples are − 242, -157, and − 115, respectively. It is noted that the values of E_ocp_ for the inhibited samples are less negative than the uninhibited sample which confirmed the ability of the extracts to inhibit the corrosion of copper in chloride medium. This performance can be explained by the adsorption through heteroatoms and active substituents (OH, SH and NH_2_) that present on the compounds of extracts (inhibitor molecules) at the surface active sites of copper^53^.

In addition, the impedance measurements are performed for copper immersed in 0.6 M NaCl without and with 60 ppm of nBF, ME, and QF samples for 3 days. The Nyquist plots are presented in Fig. 5b, the plots show that the diameter of semicircle for nBF is larger than the semicircle diameter of the blank without and with ME and QF samples indicating high resistance to corrosion and more protection. The plots are fitted to the same equivalent circuits (Fig. 4g, h) and the fitted parameters are listed in Table 9. It is noted from the values in Table 9 that all the studied samples inhibited copper corrosion even after long immersion in solution. Figure 5c & d shows that the resistance of copper decreases with time in the blank solution, however it increases with increasing time in the presence of nBF sample indicating more adsorption of nBF phytochemicals on the copper surface which blocks the active sites and reduces the dissolution of copper^5^. The molecular structure of the phytochemicals present in the studied samples plays a role in increasing the molecules adsorption and exchanging charges with copper surface to form new bonds^54,55^. In addition, the direction of the molecules and their structure in space are giving the facility to form strong bonds, as the parallel orientation is favored^56^. The substituent difference in the molecules moieties is also an important parameter, as shown from Table 1, nBF sample has high content of 9-Octadecenamide (Z) as observed from GC-MS analysis which contains both -O and -N functional groups in addition to the presence of E, E,Z-1,3,12-Nonadecatriene-5,14-diol with its -OH groups that increases the facility to be adsorbed on the copper surface.

**Table 9:** The fitted impedance parameters for copper after 72 h of immersion in 0.6 M NaCl without and with 60 ppm of the inhibitor.

Type of inhibitor	*R*_s_ (Ω)	*R*_ct_ (Ω cm^2^)	Q_ct_		*R*_ads_ (KΩ cm^2^)	Q_ct_	
			Y(µΩ^−1^cm^−2^)	*n*		Y (µΩ^−1^cm^−2^)	*n*
Blank	25.9	693	1910	0.50	0.876	45.4	0.90
nBF	26.0	1450	42.5	0.54	1.14	700	0.95
ME	10.2	4.96	2720	0.50	0.834	0.061	0.99
QF	4.83	7.31	341	0.70	0.972	878	0.95

### Surface morphology

**Fig. 6:**
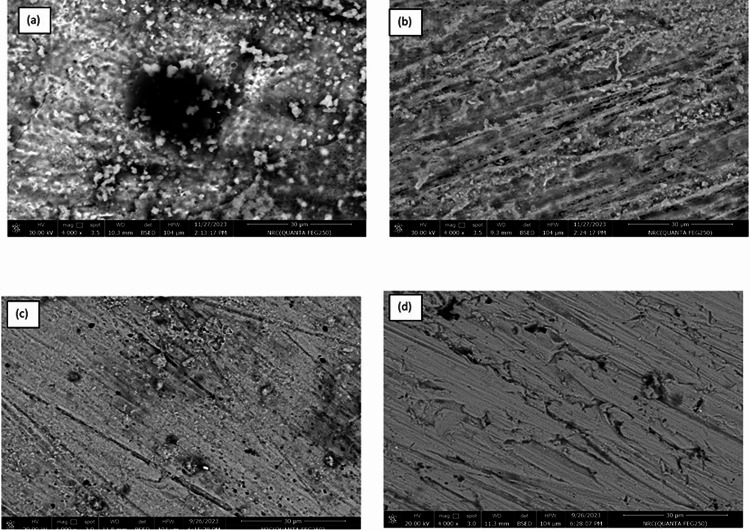
Scanning electron micrographs for copper after 3 days immersion in (**a**) 0.6 M NaCl, (**b**) 0.6 M NaCl + 60 ppm nBF, (**c**) 0.6 M NaCl + 60 ppm ME, and (**d**) 0.6 M NaCl + 60 ppm QF.

To confirm the effect of the extracts on the corrosion of copper in 0.6 M NaCl, the morphology of the four surfaces of copper after 3 days immersion in NaCl solution without (Blank) and with 60 ppm of each of nBF, ME and QF samples are studied as presented in Fig. 6a–d. The surface of copper immersed in NaCl shows the formation of an oxide film with big pores and the precipitation of some crystals from the chloride solution (Fig. 6a), however the inhibited copper surface with 60 ppm nBF sample appears homogenously without pores indicating the formation of protective adsorbed film on Cu surface as shown in Fig. 6b^57^. The inhibited copper surface by 60 ppm ME shown in Fig. 6c shows the formation of thin film where the scratched lines appear with the presence of tiny pores, however, the surface inhibited by 60 ppm QF in Fig. 6d shows a thick film with larger pore size than the ME inhibited surface. The morphology results confirm that the order by which the samples studied decrease the copper corrosion is nBF > ME > QF which consistent with the polarization and impedance results.

### MC calculations

**Fig. 7:**
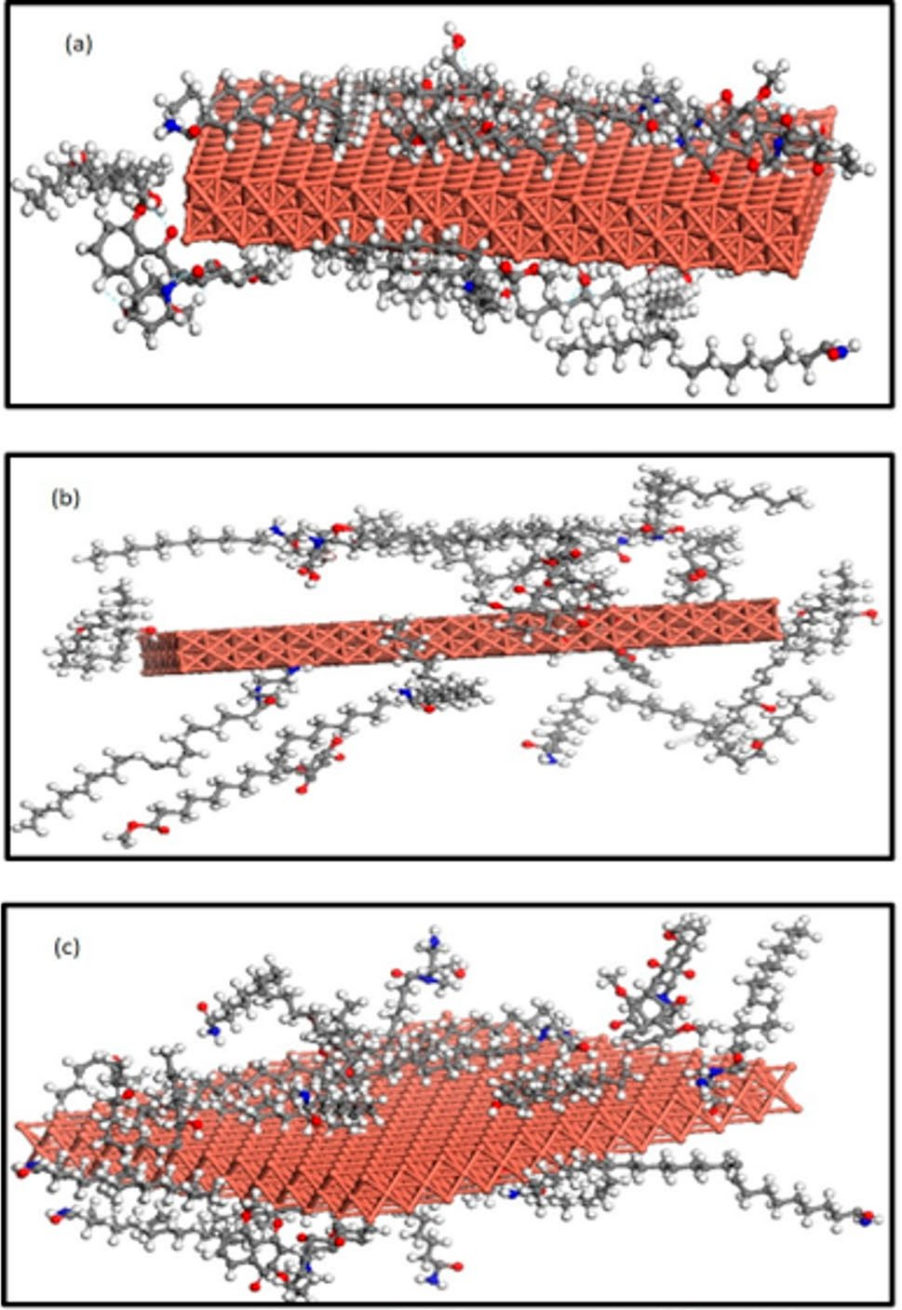
The lowest-energy structure of the interaction of fractions nBF (**a**), ME (**b**) and QF (**c**) with 10 nm copper box, as obtained from the MC simulation.

MC simulations are applied to understand the interaction between ME, QF and nBF samples with Cu surface. Figure 7a–c shows the lowest-energy structures of the interaction of the studied green samples with Cu surface, while the calculated adsorption energies (∆E_ads_) are summarized in Table 10. It is notice from the figure that, the presence of heteroatoms (O and N) significantly affects the adsorption pattern on Cu plane. This leads to interactions between the backbone of the inhibitor molecule and the surface atoms. Also, the values of the calculated ∆E_ads_ for all studied samples exhibit a notably high degree of adsorption to Cu surface, signified by their elevated ∆E_ads_ values and follows the following order ∆E_ads_ ( nBF) > ∆E_ads_ (ME) > ∆E_ads_ (QF). This intensive interaction results in the formation of a protective adsorbed layer on Cu surface, effectively acting as a barrier to impede further corrosion. As demonstrated from the figure, the observed order in the calculated values of ΔE_ads_ can be correlated to the orientation of the predominant phytochemical compounds of the studied samples on Cu surface. The parallel orientation of 9-octadecenamide compound observed in nBF, facilitates its stronger interaction with Cu surface, leading to its higher adsorption energy, compared to the perpendicular orientation in QF.

**Table 10:** The adsorption energies values of ME, QF and nBF on copper box with size 10 nm.

Samples	∆E_ads_ (kcal / mol)
ME	253.87
QF	178.97
nBF	374.56

Finally, the mechanism of inhibition of these molecules can be suggested to be due to the electrostatic adsorption of the partially negative oxygen atoms of the inhibitor molecules on the positively charged sites Cu^2+^, or the coordination of these positive ions with the heteroatoms such as N or O. These interactions can slow down the copper dissolution and shield the aggressive ions interaction (Cl^−^) resulting in forming a more protective adsorbed layer on the copper surface.

### Antimicrobial activity

**Fig. 8:**
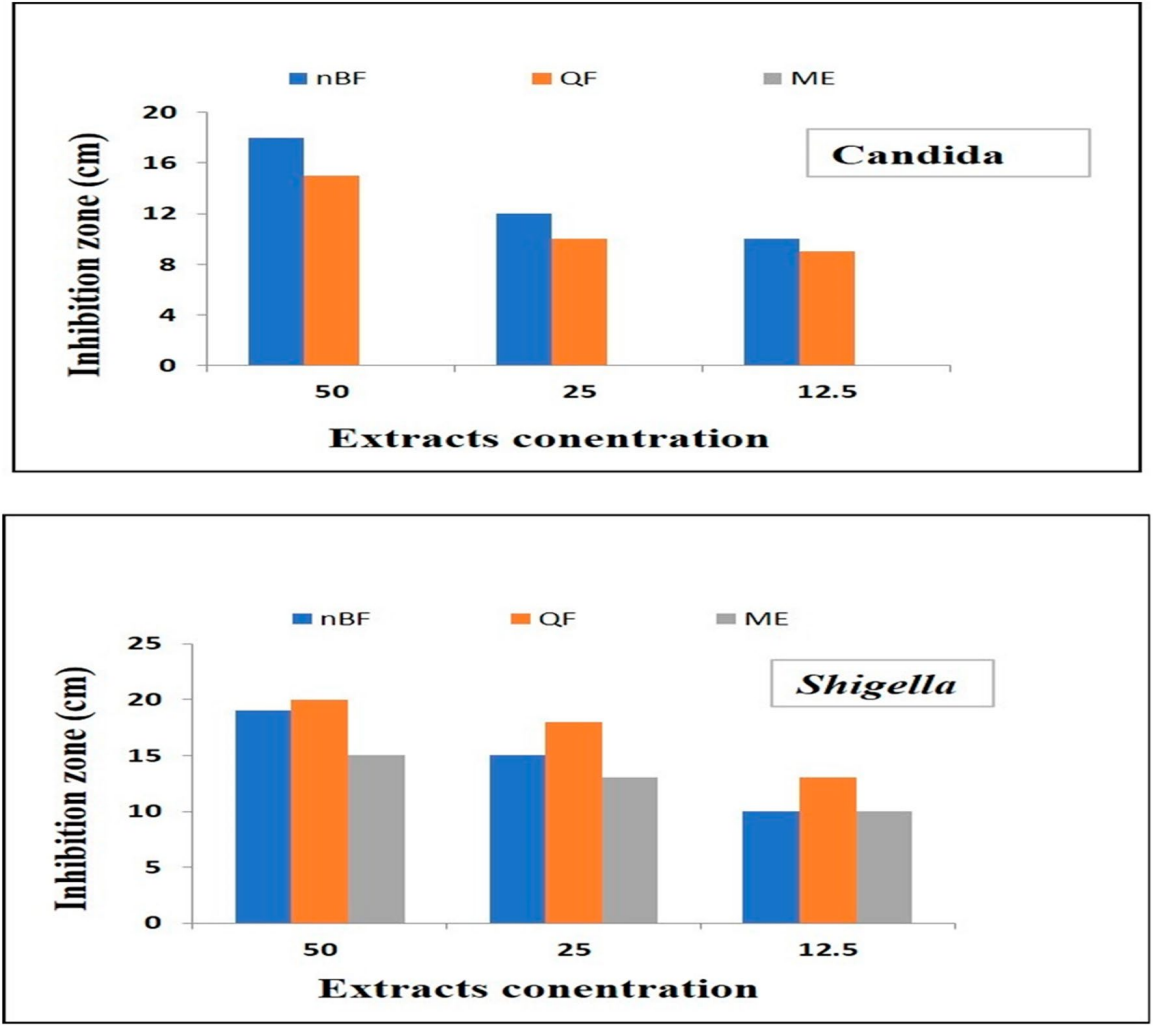
Relation between ME, Qf and nBF concentations and their inhibition activity against *Shigella* and *Candida*.

Anti-microbial activity of ME, QF and nBF samples are tested against against Staphylococcus aureus, Bacillus cereus, Listeria monocytogenes, Escherichia coli, Salmonella typhi, Shigella boydii, Aspergillus niger and Candida albicans. Results show low inhibition zone with all bacteria except Shigella and *Candiaa*. QF and nBF samples show highest antimicrobial activity against Shigella 26 and 25 cm inhibition zone, respectively, and Candida with 18 and 21 cm inhibition zone, respectively. Whereas the lower activities against Shigella and Candida are recorded by ME sample with 16 and 12 cm inhibition zone, respectively. Increasing the dilution of the studied green compounds leading to the decrease in their antimicrobial activities against Shigella and Candida. The antimicrobial and the minimum inhibition concentrations data are recorded in Tables 11 and 12 and illustrated in Fig. 8.

**Table 11:** Inhibition activity of ME, QF and nBF against Shigella and Candida.

	nBF			QF			ME		
Microorganisms	Concentration (mg\ml)								
	50	25	12.5	50	25	12.5	50	25	12.5
Shigella	Zone of inhibition (cm)								
	19	15	10	20	18	13	15	13	10
Candida	18	12	10	15	10	9	0	0	0

**Table 12:** Antimicrobial activity of ME, QF and nBF against pathogenic microorganisms.

Microorganisms	nBF	QF	ME
Zone of inhibition (cm)			
*Bacillus Cerieus*	5	5	5
*Listeria monocytogens*	5	5	5
*Staphylococcus aureus*	5	5	5
*E.coli*	5	5	5
*Salmonella typhis*	5	5	5
*Shigella boydii*	25	26	16
*Aspergillus niger*	0	0	0
*Candida albicans*	21	18	12

## Conclusion

In this study, extracted and fractionated phytochemicals of Terminalia bentzoe leaves (ME, QF, nBF) are elucidated and investigated as sustainable green inhibitors against Cu corrosion in saline solution, as well as their antimicrobial activity against a variety of bacterial infections. According to the electrochemical measurements, the compounds studied revealed as efficient anodic inhibitors for Cu in 0.6 M NaCl solution, with 94.4% maximum value of inhibition efficiency for 60 ppm of nBF sample. The FESEM analysis indicated that the inhibited surface with nBF appears homogenous without pores indicating the formation of adsorbed protective film on the surface. MC simulations confirm the adsorption of the protective layers onto the Cu surface with different orientation of the predominant phytochemical compound of the extract and its fractions. Also, the studied green compounds show good resistance against a variety of bacterial infections.

### Ethics approval

Not applicable.

## Data Availability

All data generated or analysed during this study are included in this published article.
